# Prevalence and correlates for school truancy among pupils in grades 7-10: results from the 2004 Zambia Global School-based Health Survey

**DOI:** 10.1186/1756-0500-5-48

**Published:** 2012-01-20

**Authors:** Adamson S Muula, Emmanuel Rudatsikira, Olusegun Babaniyi, Peter Songolo, Seter Siziya

**Affiliations:** 1 Department of Community Health, College of Medicine, University of Malawi, Blantyre, Malawi; 2 School of Community and Environmental Health, Old Dominion University, Norfolk, VA, USA; 3 World Health Organization Country Office, Lusaka, Zambia; 4 Department of Community Medicine, School of Medicine, University of Zambia, Lusaka, Zambia

## Abstract

**Background:**

There are limited data on the prevalence and associated factors of truancy in southern Africa. Yet truancy should attract the attention of public health professionals, educators and policy makers as it may be associated with adolescent problem behaviours. The objectives of the study were to estimate the prevalence and determine correlates of school truancy among pupils in Zambia.

**Findings:**

We used data collected in 2004 in the Zambia Global School-based Health Survey. Logistic regression analysis was conducted to identify factors associated with truancy. A total of 2257 pupils participated in the survey of whom 53.9% were male. Overall 58.8% of the participants (58.1% of males and 58.4% of females) reported being truant in the past 30 days. Factors associated with truancy were having been bullied (AOR = 1.34, 95% CI [1.32, 1.36]), current alcohol use (AOR = 2.19, 95% CI [2.16, 2.23]), perception that other students were kind and helpful (AOR = 1.12, 95% CI [1.10, 1.14]), being male and being from the lowest school grade. Pupils whose parents or guardians checked their homework (AOR = 0.91 95% CI, [0.89, 0.92]) and those who reported parental supervision (AOR = 0.94, 95% CI [0.92-0.95]) were less likely to report being truant.

**Conclusions:**

We found a high prevalence of truancy among pupils in grades 7-10 in Zambia. Interventions aimed to reduce truancy should be designed and implemented with due consideration of the associated factors.

## Background

There is growing health-related literature of truancy and its predictors from the developed world, but not much has been reported from the developing world. The potential consequences of truancy as a social problem spans across reproductive and sexual health, mental health, pulmonary health, injury experience, adult underachievement and poverty [1-4].

To explain why truancy may be associated with harmful behaviours, Henry and Huizinga [5] argued that the unsupervised and unmonitored time with peers may facilitate exposure to potentially harmful lifestyles such as illicit drug use. Olley [6] found that 46% of street youth (who lacked parental supervision) in Ibadan, Nigeria reported history of truancy.

The role of formal education in promoting health and social well-being cannot be over-emphasized. And yet the attainment of education by pupils can be disturbed by truant behaviours. We therefore carried out this study to estimate the prevalence and associated factors of school truancy among pupils in grades 7-10 in Zambia. This information is potentially useful in designing programs that may reduce the problem of truancy in the country.

## Methods

This study involved analysis of data from the Zambia Global School-based Health Survey (GSHS) that was conducted in 2004. Developed by the World Health Organization (WHO) in collaboration with UNICEF, UNESCO, and UNAIDS with technical assistance from the Centres for Diseases Control and Prevention (CDC), Atlanta, Georgia, United States, the GSHS aims to provide data on health and social behaviours among in-school adolescents. Further details of the GSHS have been reported elsewhere [7-10].

In Zambia, pupils from grades 7-10 were recruited. Although 3021 pupils were eligible to participate in the survey, only 2257 eventually participated, giving a response rate of 75%. The survey used a two-stage probability sampling technique. In the first stage of sampling, the primary sampling units were schools. These were selected with a probability proportional to their enrolment size. In the second step, a systematic sample of classes in the selected school was obtained. All pupils in the selected classes were eligible to participate.

The data are de-identified and are in public domain with global authorization for re-use. Prior to commencement of the survey, permission to carry out the study was obtained from the Ministries of Health and Education. Informed consent to participate in the study was obtained from school managers and students. Students anonymously and voluntarily completed the questionnaire.

The completion of the questionnaire occurred within one class period. Pupils were encouraged to answer all questions but also told that they were free not to answer any question they felt uncomfortable with. A total of 12 trained research assistants supervised the process. Pupils were asked: During the past 30 days, on how many days did you miss classes or school without permission? The responses were 0 days, 1 or 2 days, 3-5 days, 6-9 days and 10 or more days. A binary variable was created where a response of 0 was recoded as having never missed school or class (0) while any number of days > 0 was recoded as 1. Regarding the predictor variables, pupils were asked the following questions: During the past 30 days, how often did you go hungry because there was not enough food in your home? During the past 30 days, how often were most of the students in your school kind and helpful? During the past 30 days, how often did you parents or guardians check to see if your homework was done? During the past 30 days, how often did your parents or guardians really know what you were doing with your free time? The responses to the above predictor variables were never, rarely, sometimes, most of the time, and always. These responses were categorized into a yes (combined rarely, sometimes, most of the time and always) or a no. The responses for the other two predictor variables: "During the past 30 days, on how many days did you have at least one drink containing alcohol?" and "During the past 30 days, on how many days were you bullied?" were 0 days, 1 or 2 days, 3-5 days, 6-9 days, 10-19 days, 20-29 days and all 30 days. The responses were categorized into a yes (having been bullied for at least 1 day in the past 30 days) or a no.

### Data analysis

Data analysis was performed using SPSS version 14.0 software. The outcome variable was history of truancy. Due to the nature of the study design a weighting factor was used in the analysis to reflect the likelihood of sampling each pupil and to reduce bias by compensating for differing patterns of non response.

We obtained frequencies to describe the sample and estimate the prevalence of truancy. We conducted logistic regression in bivariate analyses and multivariate logistic regression analysis using the backward variable selection method to estimate associations between relevant predictor variables and truancy within the last 30 days. We report unadjusted odds ratios (OR) together with their 95% confidence intervals (CI) for selected predictor variables while considering having been truant in the last 30 days as a dependent variable. We also report adjusted odds ratios (AOR) and their CI from a multivariate analysis considering factors that were significantly associated with the outcome in bivariate analyses.

## Results

A total of 2257 pupils participated in the Zambian Global School-based Health Survey that was conducted in 2004. Of those whose data were available, 53.9% were male. Overall 58.8% of the participants (58.1% of males and 58.4% of females) reported being truant in the past 30 days, while 42.2% were current drinkers, and 62.8% reported having been bullied in the past 30 days. Further description of the study sample is shown in Table 1.

**Table 1:** Socio-demographic and truancy characteristics of pupils in grades 7-10 in Zambia, 2004

	Total	Male	Female
Factor	n* (%)**	n* (%)**	n* (%)**
Age			
< 14	463 (27.5)	156 (21.5)	263 (30.9)
14	386 (19.1)	156 (17.4)	219 (21.6)
15	513 (22.8)	260 (24.5)	238 (21.6)
16 +	708 (30.6)	394 (36.5)	306 (25.9)
Sex			
Male	994 (53.9)	-	-
Female	1039 (46.1)		
Grade			
7	840 (56.5)	344 (55.1)	463 (57.3)
8	552 (20.7)	291 (21.0)	241 (20.4)
9 or 10	670 (22.8)	343 (23.9)	317 (22.3)
Hungry			
Yes	1691 (82.5)	787 (82.3)	834 (83.4)
No	366 (17.5)	173 (17.7)	170 (16.6)
Peers kind and helpful			
Yes	1292 (74.4)	592 (73.2)	636 (75.9)
No	452 (25.6)	222 (26.8)	210 (24.1)
Parents checked homework			
Yes	1339 (74.5)	602 (73.1)	679 (77.0)
No	448 (25.5)	224 (26.9)	197 (23.0)
Parental supervision			
Yes	1369 (77.4)	634 (78.1)	673 (77.2)
No	377 (22.6)	169 (21.9)	189 (22.9)
Bullied			
Yes	949 (62.8)	431 (60.0)	466 (65.0)
No	610 (37.2)	314 (40.0)	274 (35.0)
Current alcohol drinking			
Yes	528 (42.2)	217 (38.5)	283 (45.1)
No	805 (57.8)	388 (61.5)	388 (54.9)
Missed classes or school			
Yes	1010 (58.8)	365 (58.1)	381 (58.4)
No	772 (41.2)	463 (41.9)	493 (41.6)

Frequencies not adding up because of missing information

* unweighted frequencies

** weighted percents

In bivariate logistic regression analyses (Table 2), we found that pupils in the youngest age group < 14 years were less likely to be truant compared to pupils who were aged 16 years or older. However, pupils who were in the middle age categories were more likely to be truant. In other results of bivariate analyses we found that current alcohol use, having been bullied, perception that peers were helpful and kind and having gone hungry because of lack of food at home were associated with truancy. Gender and parental supervision were not associated with truancy, while parental support with homework was negatively associated with the outcome.

**Table 2:** Factors associated with truancy among pupils in grades 7-10 in Zambia, 2004

Factor	OR* (95%CI**)	AOR*** (95%CI)
Age		
< 14	0.90 (0.88, 0.91)	0.67 (0.65, 0.68)
14	1.15 (1.13, 1.16)	1.08 (1.05, 1.11)
15	1.04 (1.03, 1.06)	1.12 (1.09, 1.15)
16 +	1	1
Sex		
Male	1.00 (0.99, 1.00)	1.16 (1.14, 1.18)
Female	1	1
Grade		
7	1.21 (1.19, 1.22)	1.41 (1.38, 1.44)
8	1.03 (1.02, 1.04)	0.92 (0.90, 0.94)
9 or 10	1	1
Hungry		
Yes	1.17 (1.16, 1.18)	-
No	1	
Peers kind and helpful		
Yes	1.02 (1.01, 1.03)	1.12 (1.10, 1.14)
No	1	1
Parents checked homework		
Yes	1.00 (0.99, 1.01)	0.91 (0.89, 0.92)
No	1	1
Parental supervision		
Yes	0.95 (0.94, 0.96)	0.94 (0.92, 0.95)
No	1	1
Bullied		
Yes	1.82 (1.80, 1.84)	1.34 (1.32, 1.36)
No	1	1
Current alcohol drinking		
Yes	2.52 (2.49, 2.54)	2.19 (2.16, 2.23)
No	1	1

OR* Unadjusted odds ratio; CI** Confidence Interval; AOR*** Adjusted odds ratio for all the significant factors in a multivariate analysis

In a multivariate analysis (Table 2), age, sex, school grade, perception that other students were kind and helpful, history of being bullied, alcohol use, perception that peers were helpful, parents or guardians checked their homework, parental supervision, and having been bullied were significantly associated with truancy.

Participants who were bullied (AOR = 1.34, 95% CI [1.32, 1.36]), consumed alcohol (AOR = 2.19, 95% CI [2.16, 2.23]), perceived that other students were kind and helpful (AOR = 1.12, 95% CI [1.10, 1.14]), male and from the lowest school grade were more likely to report being truant. Pupils whose parents or guardians checked their homework (AOR = 0.91 95% CI, [0.89, 0.92]) and those who reported parental supervision (AOR = 0.94, 95% CI [0.92-0.95]) were less likely to report being truant.

## Discussion

We found the prevalence of truancy within the last 30 days to be 58.8% among pupils in grades 7-10 in Zambia. This rate was much higher than what we found in Swaziland of 21.6% [11]. In a study of 8th and 10th graders in the United States, Henry reported that 11% of the former and 16% of 10th graders reported recent truancy in past 4 weeks [12]. The high rate of truancy in this study may be due to the high rate of alcohol use (42.2%) compared to 16.6% in Swaziland [11]. It is also possible that adolescents from poor households may miss school to work at home or elsewhere.

Like in other studies, we found that males were at increased likelihood of being truant than females. Siziya et al. [11] reported that, in Swaziland, male adolescents were more likely to be truant than females. Likewise in United States, Weden and Zabin [13] reported that males were more likely to engage in problem behaviours, including being truant among adolescents in the National Longitudinal Survey of Youth (1997-2000). We also found that pupils who were of ages less than 14 years were less likely to be truant than older pupils. Younger pupils are more likely to be under parental supervision than older pupils and may thus be less likely to be truant than older pupils. The finding on the association of grade with truancy was inconsistent. While pupils in grade 7 were more likely to be truant than those in grades 9 or 10, those in grade 8 were less likely to be truant. It is inconceivable that pupils in examination grades (grades 7 and 9) could be more likely to be truant than those in a non examination grade 8 when they should not be missing classes to prepare for their examinations. We offer no explanation for this observation.

In our study, pupils who felt that their peers were kind and helpful were more likely to be truant. In a study of school-related risk and protective factors associate with truancy among urban youth placed at risk, Henry and Huizinga (2007) found that having delinquent peers was a strong predictor of truancy [14]. It is possible that truant students, who constitute the majority of the respondents in this study, may be kind and supportive to those who are involved in the same behaviour.

We found that pupils who reported that their parents or guardians checked their homework or reported that they received parental supervision were less likely to be truant. In a randomized controlled trial, Stanton et al. [15] found that parental involvement with and support of the adolescent was associated with less truancy and other problem behaviours. Henry [12] also found that a large amount of unsupervised time was associated with truancy. Parental supervision is an important determinant of truancy in many settings [11,15]. In another study by Miller and Plant [16], however, parental caring and control were not significantly associated with truancy.

We also found that study participants who reported being victims of bullying were more likely to have been truant. It is possible that adolescents who are bullied may miss school in order to escape further victimization. While truancy is a problem behaviour on its own, it is important that parents and school authorities explore the reasons why a pupil may be missing school without permission. A bully-free school may reduce the prevalence of truancy [17,18].

Alcohol consumption was significantly associated with truancy in the current study. This finding was similar to that previously reported from Swaziland [11]. Best et al. [3] have also reported among 14-16 year olds in the United Kingdom that excessive alcohol drinking was associated with frequent truancy. In that study excessive drinking was defined as drinking 10 or more units of alcohol on one occasion.

Our study had a few limitations. Firstly the study was based on data based on self reports. Pupils may have misreported either due to failure to recall or misreported intentionally. Completion of the study questionnaire was done anonymously so as to maintain confidentiality and reduce possibility of intentional misreporting. Also data were collected from pupils who were available in school on the day of the survey. Pupils who were absent being both truant and otherwise were not included, and thus we may have underestimated the prevalence of truancy. Although the responses to the predictor variables were obtained as categories, we further dichotomized them, and this may have reduced the statistical power in the analysis.

## Conclusions

Our study found the prevalence of school truancy within the past 30 days among pupils in grades 7-10 in Zambia to be 58.8%. Factors such as current alcohol drinking and being bullied, which have been reported elsewhere as predictors of truancy, were also identified in our study. Designing of intervention programs aimed to reduce truancy in Zambia should consider these factors.

## Competing interests

The authors declare that they have no competing interests.

## Authors' contributions

ASM participated in the interpretation of the data and led the drafting of the manuscript. ER helped in data analysis and interpretation and revision of the manuscript. OB and PS were involved in drafting the manuscript or revising it critically for important intellectual content. SS carried out data analysis and participated in the drafting of the manuscript. All authors read and approval the final manuscript.
